# Dietary Supplementation of *Limosilactobacillus mucosae* LM1 Enhances Immune Functions and Modulates Gut Microbiota Without Affecting the Growth Performance of Growing Pigs

**DOI:** 10.3389/fvets.2022.918114

**Published:** 2022-06-30

**Authors:** Qianqian Zhang, Robie Vasquez, Jeong Min Yoo, Sang Hoon Kim, Dae-Kyung Kang, In Ho Kim

**Affiliations:** Department of Animal Resources and Science, Dankook University, Cheonan, South Korea

**Keywords:** growing pig, nutrition digestibility, immune function, gut microbiota community, intestinal morphology, short-chain fatty acid

## Abstract

*Limosilactobacillus mucosae* LM1 (LM1) is previously isolated from the intestine of piglets, but its potential as a probiotic supplement has not yet been assessed in growing pigs. In this study, we analyzed the probiotic effect of LM1 on the growth performance, apparent total tract digestibility (ATTD) of nutrients, immune properties, intestinal morphology, and gut microbiota and their metabolites in growing pigs. The experiment included 145 Duroc × (Landrace × Yorkshire) pigs (average body weight: 21.21 ± 1.14 kg) distributed into five treatment groups. The pigs were fed either a control diet (CON), or the control diet supplemented with incremental doses of LM1, namely low-dose LM1 (LL, 8.3 × 10^8^ CFU/kg), moderate-low dose LM1 (ML, 4.2 × 10^9^ CFU/kg), moderate-high dose LM1 (MH, 8.3 × 10^9^ CFU/kg), and high-dose LM1 (HH, 2.1 × 10^10^ CFU/kg) for 42 d. On d 42, 12 pigs from each of the CON and MH groups were slaughtered. The results indicated that the ATTD of nitrogen (N, *P* = 0.038) was improved with MH supplementation. In addition, increasing dose of LM1 improved the immune response in pigs by reducing serum pro-inflammatory cytokines (interleukin-1β and tumor necrosis factor-alpha) and increasing anti-inflammatory cytokines (interleukin-10). Pigs fed with MH LM1 also had higher jejunal villus height and ileal villus height: crypt depth ratio, demonstrating improved intestinal morphology. Moreover, moderate-high LM1 supplementation enriched SCFA-producing taxa such as *Lactobacillus, Holdemanella, Peptococcus, Bifidobacterium, Eubacterium_hallii*_group, and *Lachnospiraceae_AC2044_*group, which correlated positively with increased fecal levels of butyrate and iso-valerate. These results strongly suggest the probiotic potential of LM1 on growing pigs. Overall, the current study provides insights on the use of *L. mucosae* LM1 as a novel livestock probiotic to improve pig gut health.

## Introduction

Absorbing nutrients and resisting external interferences are vital functions of the gastrointestinal tract (GIT). The entry of mycotoxins via feed ingredients is inevitable and they trigger damage to the intestinal mucosa, leading to an inflammation response (www.biomin.net). Moreover, any changes in the feed composition or environment contribute to a short or long-term variation in intestinal microbiota (1). Such changes pose a risk to the health of pigs during their rapid growth phase and may restrict their growth performance.

Probiotics is defined by FAO and WHO as “live microorganisms” (2), reportedly, they are known to promote growth (3, 4), stimulate the production of digestive enzymes, regulates gut microbiome, improves immune response, and protects intestinal barrier in pig production (5). Among them, *Lactobacillus* is a predominant genus in growing pigs (6), and its strains are commonly used as probiotics. Evidence indicates that consumption of *L. reuteri* LR1, *L. salivarius*, and *L. casei* can boost the growth of broilers and weaned piglets (4, 6, 7) by improving digestibility, modulating gut microbiota, or improving the villus height and immune status. Interestingly, the majority of *Lactobacillus*, such as *L. reuteri, L. salivarius*, and *Lm. mucosae* can adhere to the surface of the mucosal membrane of the GIT, thereby suppressing the colonization of *Salmonella* and *Escherichia coli in vivo* and *in vitro* (6, 8). Additionally, supplementation with *Lactobacillus* spp. can enhance the intestinal barrier by downregulating pro-inflammatory cytokines such as interleukin-1β (IL-1β), interleukin-6 (IL-6), and tumor necrosis factor-alpha (TNF-α) and increasing the mRNA levels of tight junction proteins (9, 10). Additionally, *Lm. mucosae* isolated from the fecal matter of donkey has exhibited antioxidant properties *in vitro* (11). Supplementation of *Lactobacillus* strains also affects the gut microbiome metabolites, namely the short-chain fatty acid [SCFA; (12)]. However, the characteristics and capacities of specific *Lactobacillus* strains are inconsistent.

*Limosilactobacillus mucosae* LM1 (LM1, formerly *Lactobacillus mucosae* LM1), a member of family Lactobacillaceae (13), was isolated from the intestine of healthy piglets (14). Genome analysis has revealed that LM1 possesses a gene encoding a mucus-binding protein, thereby enabling it to adhere to the mucosal surface of the intestine and protect the cell surface from antimicrobial agents (13). An *in vitro* study by Valeriano et al. (8) has revealed that the properties of LM1 can help to protect the host against pathogens such as *E. coli* and *S. typhimurium*, survive in bile with a low pH of 3, and produce beta-galactosidase enzyme. These characteristics imply that LM1 can reach the intestine and inhibit colonization of the ecological locus of pathogenic bacteria. Although LM1 has been studied extensively *in vitro*, its probiotic ability *in vivo* is yet to be investigated. Therefore, this trial was performed to assess the influence of LM1 on the growth performance, nutrient digestibility, gut microbiota, and SCFA metabolism of growing pigs.

## Materials and Methods

### Ethics and Approval

This experiment was conducted at the swine experimental base in Cheonan City (South Korea). All experimental and animal management procedures were implemented according to the Guide of Laboratory Animals provided by the Institutional Animal Care Advisory Committee for Dankook University (Protocol number: DK-1-2104). The entire study design was approved by the Animal Management Committee of the Dankook University, South Korea.

### Animals, Feed, and Management

The experiment consisted of 145 Duroc × (Landrace × Yorkshire) growing pigs (72 male: 73 female) with an average body weight (BW) of 21.42 ± 1.14 kg. The pigs were divided into five treatment groups with six repeat pens (29 pigs / treatment, two barrows: three gilts or two barrows :two gilts per pen) throughout the 42-d trial, based on a randomized block design. The five treatment groups were fed on five different diets, namely basal diet without any additives (CON) or basal diet supplemented with LM1 at different doses: low-dose LM1 (LL, 8.3 × 10^8^ CFU/kg), moderate-low dose LM1 (ML, 4.15 × 10^9^ CFU/kg), moderate-high dose (MH, 8.3 × 10^9^ CFU/kg), and high-dose LM1 (HH, 2.1 × 10^10^ CFU/kg). The ingredients and their respective nutritional values in the basal diet, as shown in Table 1, were formulated to meet the recommendations of the National Research Council (15) for pigs weighing 25–50 kg. During the 42-d trial period, the pigs were provided with water and feed *ad libitum*. Internal room temperature of 24°C and air humidity of 50–60% were maintained by an automatic environmental control device.

**Table 1:** Ingredients and nutrient level of basal diet (as-fed basis).

**Ingredient**	**%**
Corn	66.90
Soybean meal	23.69
Tallow	3.11
Molasses	3.00
Limestone	1.18
Monodicalcium phosphate	0.93
Salt	0.40
L-met (98%)	0.05
L-Lys (78%)	0.32
L-Thr (98.5%)	0.10
L-Trp (98%)	0.10
Vitamin-mineral Premix^a^	0.20
Choline	0.02
Total	100.00
**Calculated nutrient value, 100%**	
Metabolizable energy, Mcal/kg	3.35
Crude protein	16.00
Crude fat	5.63
Crude fiber	2.89
Crude ash	4.42
Ca	0.66
Total P	0.51
Available P	0.26
SID lysine	0.98
SID methionine	0.28
Methionine + cystine	0.56
Threonine	0.59
Tryptophan	0.17

^a^*Provided per kg of complete diet: 6,000 IU vitamin A, 800 IU vitamin D3, 40 IU vitamin E, 2.0 mg vitamin K, 4.0 mg vitamin B6, 3.0 mg vitamin B12, 20 μg pantothenic acid, 15 mg niacin, and 0.02 mg biotin; 150 mg Cu (CuSO_4_ ·5H_2_O), 150 mg Fe (FeSO_4_), 50 mg Zn (ZnSO_4_), 0.5 mg I (KI), 2 mg Mn (MnSO_4_·H_2_O), and 0.3 mg Se (Na_2_SeO_3_)*.

### Preparation of Probiotics

*Lm. mucosae* LM1 was previously isolated in healthy pigs by Lee et al. (14). Identification, characterization, and evaluation of the probiotic potential of LM1 were done previously in our laboratory by Lee et al. (14) and Valeriano et al. (8). The genome sequence of LM1 was deposited in NCBI Genbank under accession number AHIT00000000 (14). For the feeding trial, pure culture LM1 was cultivated in de Man-Rogosa-Sharpe broth (Difco, Pont-de-Claix, France) at 37°C for 48 h, then centrifuged at 5,000 × *g* at 4°C to recover cell pellet. It was then processed into powdered form by freeze-drying (Eyela Co., Japan) at – 60°C for 24 h, then mixed with the basal feed to achieve their individual dosages. These processes were performed in Sunbio Ltd. (Cheonan, South Korea). Feed mixed with probiotics were kept in a sterile container, at 4°C. LM1 viability was maintained above 95% for 6 weeks prior feeding (data not shown).

### Sample Collection

The impact of LM1 on nutrient absorption and digestibility in pigs was evaluated in the following manner. From d 36 to 42, of the trial period, chromium trioxide (Cr_2_O_3_) was added to the diet at a dose of 2.5 g/kg of feed. From d 39 to 42, fecal samples were collected from 2 pigs/pen by stimulating their anal sphincters. Thereafter, the fecal samples were mixed proportionally, and the feed and fecal were frozen at −20°C for further analyses. Thawed feeds and fecal samples were weighed and subsequently baked at 72°C for 60 h until a constant weight was achieved. Ultimately, the fecal samples were ground and sieved through a 40-mesh screen, while the ground feed samples were passed through a 25-mesh screen. The consequent assays and the instruments used for measuring dry matter (DM), N, and gross energy (GE) of the samples (feed and fecal) were according to a previous study by Zhang et al. (16). The apparent total tract digestibility (ATTD) of nutrients was calculated using the formula, ATTD (%) = [1 – {(Nf × Cr_2_O_3_d) / (Nd × Cr_2_O_3_f)}] × 100, where Nf and Nd represent the nutrient concentrations and Cr_2_O_3_f and Cr_2_O_3_d represent the chromium trioxide concentrations in feces and diet, respectively. All these values are presented as percentages of the total dry matter.

On the morning of d 42, individual BWs of the pigs (after 8 h of starvation) were measured. Additionally, the total feed input and residual feed were calculated on a pen-to-pen basis to estimate the average daily gain (ADG), average daily feed intake (ADFI), and conversion ratio (G/F) of pigs. Immediately after BW measurement, blood samples (5 mL /pig) were collected in dipotassium ethylenediaminetetraacetic acid (EDTA-K2) vacuum tubes from the jugular vein of one randomly selected pig /pen. Thereafter, the serum was harvested by centrifuging blood samples at 4,000 × for 10 min and stored at −20°C until further analysis.

Individual backfat thickness of each pig was measured at the 10th rib (6 cm from the midline) using an ultrasound instrument (Piglot 105; SFK Technology, Herlev, Denmark) at the beginning and end of the trial.

Thereafter, 24 randomly selected pigs, 12 each from the CON and MH groups, were slaughtered via electrical stunning and exsanguination. Their intestines were stripped from the mesentery and immediately placed on ice. An ~2-cm long segment of the middle jejunum was cleaned with ice-cold physiological saline and placed in 4% formalin fixative for morphometric measurements. Approximately 20 cm of the remaining jejunal tissue was cut longitudinally and washed with physiological saline; consequently, the mucosa was scraped out using a sterilized slide, which, in turn, was stored at −80°C until further analysis. Prior slaughtering, fecal samples were collected in sterile tubes from each pig, placed rapidly in liquid nitrogen, and subsequently transferred to a refrigerator to be stored at −80°C for further next generation sequencing (NGS) and SCFA determination.

### Assay of Serum Cytokine Concentrations

The serum samples were analyzed for IL-1β (PLB00B), IL-6 (P6000B), TNF-α (PTA00), and interleukin-10 (IL-10, P1000) using the porcine enzyme-linked immunosorbent assay (ELISA) kits (R & D Systems, Minneapolis, MN, USA) strictly according to the instructions. Parallel determination assays were performed on each 96-well-plate for every sample, and the optical density was read using a microplate reader (SpectraMax190, MD, USA). The cytokine concentrations were calculated based on a standard curve constructed independently. Coefficients of inter-sample variations for IL-1β, IL-6, TNF-α, and IL-10 were 7, 7, 9, and 7% respectively, while intra-sample coefficients of variation for these cytokines were 6, 5, 6, and 5%, respectively.

### Assay for Antioxidant Indices in the Intestinal Mucosa

Each mucosa sample (0.1 g) was homogenized in a 9-fold volume of cold phosphate buffered saline solution and centrifuged for 10 min at 8,000 × *g*. The supernatants were collected and utilized for detecting the total antioxidant capacity (T-AOC), thiobarbituric acid reactant substances (TBARS), and protein concentration in the jejunum and ileum, according to the reagent guide (Catalog: abx298877, abx097981, abx293001; Abbexa Co., Ltd, 181 Cambridge Science Park, UK). All samples were detected in duplication, and the coefficient of variations were controlled <10% (intra-assay) and <12% (inter-assay), respectively, for 2 indices.

### Histomorphological Analysis

The fixed intestinal segments (jejunum and ileum) were rinsed for 30 min under running water and subsequently dehydrated with absolute ethanol at varying concentrations. The tissues were cleared with xylene, embedded in wax, and sliced into 5 μm-thick slices using a Leica RM2235 microtome (Leica, Germany). Finally, these tissue slices were dewaxed and subjected to hematoxylin-eosin staining. For each well-oriented villus, 10 measurements were recorded for both villus height (VH) and crypt depth (CD) using Image Pro Plus 6.0. The average of these 10 measurements was used to represent the VH and CD for each tissue. The V/C ratio was calculated by dividing the VH value by CD value.

### Determination of SCFA Concentrations

The concentrations of lactate, acetate, propionate, butyrate, isovalerate, and valerate in the fecal samples were determined, according to the protocol described by Slizewska and Chlebicz (17). Firstly, 0.5 g of fecal sample (CON, *n* = 12; MH, *n* = 11, because one piglet's fecal sample was lost) was diluted in 1 mL of sterile demineralized water and centrifuged at 15,000 × *g* for 15 min at 4 °C. The supernatant was collected, and 1 mL of it was mixed with 200 μL of 25% metaphosphoric acid and 23.3 μL of 210 mmol/L crotonic acid. The resultant solution was again centrifuged at 15,000 × *g* for 10 min at 4 °C. The supernatant was collected, and 300 μL of it was homogenized with 900 μL carbinol, followed by filtration through a 0.22-μm polytetrafluoroethylene syringe filter. Subsequently, 10 μL of the filtrate was injected into a 1,260 high performance liquid chromatography (HPLC) system (Agilent, USA) with a 300 × 7.8 mm Aminex HPX-87H column (Bio-Rad, USA) and refractive index and ultra-violet detectors (λ = 210 nm). The mobile phase was 0.005M H_2_SO_4_ with a flow rate of 0.6 μL/min. During the 35 min reaction time, the column temperature was maintained at 65 °C.

### 16S Ribosomal RNA (rRNA) Sequencing

Microbial DNA was extracted from the fecal samples (CON group, *n* = 12; MH group, *n* = 11) using QIAamp PowerFecal Pro DNA Kit (Qiagen, Hilden, Germany), according to the manufacturer's guidelines. The concentration and purity of each DNA sample were determined using a UV spectrophotometer (Mecasys Co., Ltd., Daejeon, Republic of Korea), and the DNA quality was confirmed via 2% agarose gel electrophoresis. The augmentation of the V3–V4 hypervariable region of the 16S rRNA was performed at ChunLab, Inc. (Seoul, Republic of Korea), and the high-throughput sequencing was conducted using Illumina MiSeq platform. Raw sequence data generated by the 16S rRNA gene were processed using Quantitative Insights Into Microbial Ecology pipeline [QIIME2, (18)]. Primers and adapters were removed from the raw sequences using the ‘cutadapt' plugin (19). The sequence quality control and feature table construction were performed using the divisive amplicon denoising algorithm [DADA2, (20)], and operational taxonomic units (OTUs) were constructed according to the concept of amplicon sequence variants. The feature classifiers were trained by “q2-feature-classifier” within QIIME2, using SILVA 138_99 database (21). Statistical analyses of alpha and beta diversity were performed in the QIIME2 pipeline. Differential taxonomic markers for each group were determined using linear discriminant analysis effect size [LEfSe, (22)].

### Statistical Analysis

Data were checked for normal distribution using the Shapiro-Wilk normality test prior to analysis. The growth performance, backfat thickness, and ATTD were analyzed using MIXED procedure of SAS (SAS 9.4 Institute Inc., Cary, NC) with an individual repeat pen taken as a statistical. The statistical model was as follows: Y_ij_ = μ + T_i_ + e_ij_, where Y_ij_ is the independent variable, μ is the overall mean, T_i_ is the fixed effect of the treatment, and e_ij_ is the random error associated with the *i*th treatment. Linear, quadratic, and cubic effects were analyzed using polynomial orthogonal contrasts. The coefficients for the polynomial orthogonal contrasts under an unequally spaced dose gradient were calculated using PROC OPC procedure of SAS. The PDIFF test was used for the multiple treatment comparisons. A generalized linear model was used for analyzing the intestinal mucosal morphology, antioxidant properties, and SCFA concentrations, with an individual pig taken as an experimental unit. All analytical procedures were compared using Tukey's multiple range test.

The microbial compositions were analyzed using R program (ver. 4.0.2) with Student's *t*-test comparisons. Permutational multivariate analysis of variance was used to determine significance in the principal coordinate analysis (PCoA) plot. Correlations between microbiota and SCFA concentrations were analyzed using Spearman's correlation coefficient and visualized using “Hmisc” and “heatmap” packages, respectively. Statistical significance was set at *P* < 0.05. *, **, and *** represent *P* < 0.05, 0.01, and 0.001, respectively.

## Results

### Effect of Dietary LM1 on the Growth Performance and Backfat Thickness of Growing Pigs

The effects of LM1 supplementation on the growth and backfat thickness of the pigs are shown in Table 2. After the 42-d feeding trial was complete, it was revealed that supplementation of LM1 in the diet did not induce any linear or quadratic responses (*P* > 0.05) in BW, ADG, ADFI, and G: F, as well as in the backfat thickness regardless of dose.

**Table 2:** Effects of dietary LM1 on growth performance and backfat thickness of growing pigs.

**Items**	**Dose of LM1**					**SEM^***a***^**	***P*** **-value**		
	**CON**	**LL**	**ML**	**MH**	**HH**		**Linear**	**Quadratic**	**Cubic**
**BW, kg**									
Initial	21.21	21.21	21.20	21.20	21.21	1.14	-	-	-
Final	46.38	46.50	47.46	48.99	46.52	2.91	0.699	0.918	0.737
**Overall**									
ADG, g/d	599	602	625	668	600	44	0.514	0.865	0.563
ADFI, g/d	1,335	1,341	1,377	1,403	1,330	121	0.816	0.983	0.754
G: F	0.451	0.453	0.472	0.482	0.450	0.02	0.479	0.983	0.326
**Backfat thickness, mm**									
Initial	5.60	5.62	5.62	5.64	5.62	0.09	0.801	0.990	0.821
Final	9.72	10.14	10.28	10.52	9.81	0.23	0.115	0.314	0.263

^a^*SEM represented pooled standard error of mean, n = 6*.

### Effect of Dietary LM1 on the Apparent Total Tract Digestibility of Growing Pigs

The supplementation of food with MH led to a significant elevation of (cubic effect, *P* = 0.038) the ATTD of N but did not affect the ATTD of DM or GE (Table 3).

**Table 3:** Effects of dietary LM1 on ATTD of growing pigs (%).

**Items**	**Dose of LM1**					**SEM^***a***^**	***P*** **-value**		
	**CON**	**LL**	**ML**	**MH**	**HH**		**Linear**	**Quadratic**	**Cubic**
DM	76.97	76.17	76.57	76.22	77.14	1.17	0.159	0.699	0.105
N	67.38	66.07	69.13	70.08	67.01	1.11	0.295	0.423	0.038
GE	77.32	76.31	75.27	78.55	78.26	1.45	0.691	0.156	0.399

^a^*SEM represented pooled standard error of mean, n = 6*.

### Effect of Dietary LM1 on the Serum Cytokine Concentrations of Growing Pigs

Dietary interventions using LM1 at the ML, MH, and HH levels led to a significant decrease in the IL-1β (linear, quadratic, and cubic effect, *P* < 0.05) as well as TNF-α concentrations (linear and cubic effects, *P* < 0.001). In contrast, the ML, MH, and HH supplementation significantly increased the IL-10 concentration (linear and quadratic, and cubic effect, *P* < 0.01). However, IL-6 and growth hormone concentrations were similar between the two groups (Table 4).

**Table 4:** Effects of dietary LM1 on serum cytokines of growing pigs.

**Items**	**Dose of LM1**					**SEM^**a**^**	***P*** **-value**		
	**CON**	**LL**	**ML**	**MH**	**HH**		**Linear**	**Quadratic**	**Cubic**
IL-1β, pg/mL	123.53	122.15	115.38	112.00	113.67	1.51	<0.001	0.021	0.078
IL-6, pg/mL	820	815	817	812	820	2.40	0.322	0.372	0.790
IL-10, pg/mL	171.00	167.83	177.67	182.33	173.33	1.57	0.001	0.016	0.001
TNF-α, pg/mL	322.17	328.17	306.50	298.50	326.33	2.28	<0.001	0.124	<0.001
Growth hormone, μg/L	17.30	17.23	17.82	17.70	17.53	0.22	0.159	0.699	0.105

^a^*SEM represented pooled standard error of mean, n = 6*.

### Effect of Dietary LM1 on the Intestinal Morphology and Oxidative Status and Antioxidant Indices in the Mucosa of Growing Pigs

**Figure 1:**
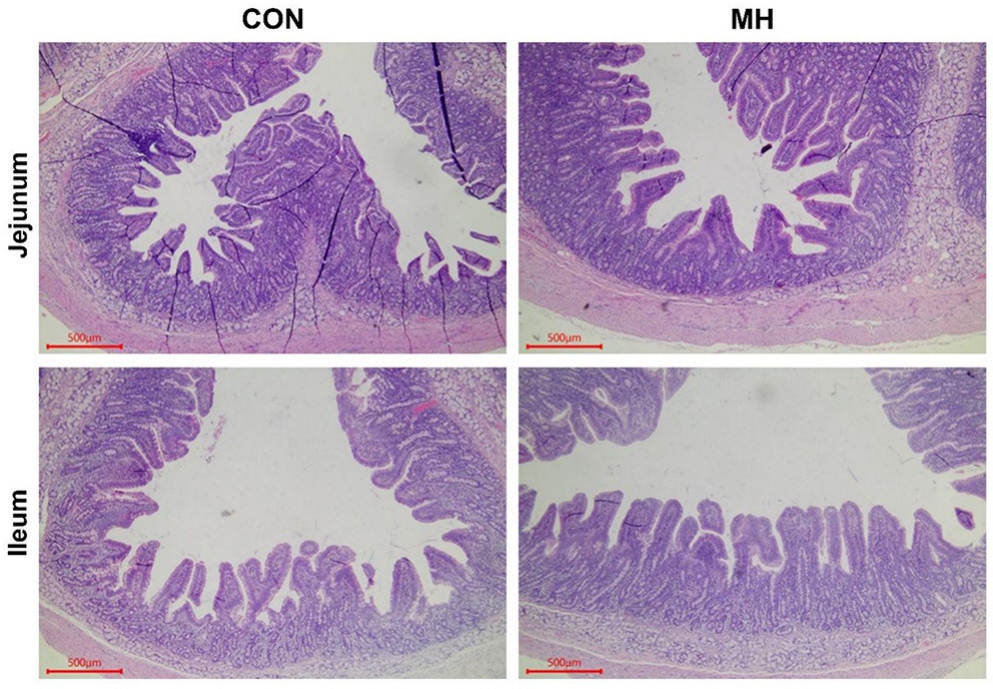
Haematoxylin and eosin (H&E) staining of jejunum and ileum. Note: Scale bar, 500 μm. CON = basal diet without any additives, MH = basal diet + 8.3 × 10^9^ CFU/kg LM1, *n* = 12 in the CON group, *n* = 11 in the MH group.

In comparison to the jejunal morphology of the CON group, the MH treatment group of pigs exhibited a significant increase (*P* = 0.012) in the VH, but no change (*P* > 0.05) in the CD or V/C ratio (Table 5, Figure 1). Moreover, the ileal VH tended to increase (*P* = 0.051), while the ileal V/C ratio was significantly increased (*P* = 0.032) in the pigs fed on MH-supplemented diet, as compared to that in the CON group. With respect to the antioxidant indices, TBARS tended to reduce (*P* = 0.063) in the ileal mucosa of the pigs fed on MH-supplemented diet, as compared to that in the CON group. Interestingly, there were no variances (*P* > 0.05) in the T-AOC or TBARS concentrations in the jejunal mucosa between the two groups (Table 6).

**Table 5:** Effects of dietary moderate-high dose LM1 on intestinal morphology of growing pigs.

**Items**	**CON**	**MH**	**SEM^***a***^**	***P*-value**
**Jejunum**				
VH, μm	489.59	502.12	2.94	0.012
CD, μm	213.85	220.08	2.68	0.128
V/C ratio	2.291	2.287	0.024	0.899
**Ileum**				
VH, μm	399.95	406.21	2.02	0.051
CD, μm	207.18	200.79	2.58	0.107
V/C ratio	1.933	2.027	0.027	0.032

^a^*SEM represented pooled standard error of mean, n = 12*.

**Table 6:** Effects of moderate-high dose LM1 on T-AOC and TBARS of colonic mucosa in growing pigs.

**Items**	**CON**	**MH**	**SEM^***a***^**	***P*-value**
**T-AOC, U/mg prot**				
Jejunum	0.68	0.74	0.03	0.231
Ileum	0.91	0.82	0.06	0.309
**TBARS, nmol/mg prot**				
Jejunum	1.83	1.90	0.03	0.150
Ileum	1.65	1.57	0.03	0.063

^a^*SEM represented pooled standard error of mean, n = 12*.

### Effect of Dietary LM1 on the Gut Microbiome Structure of Growing Pigs

**Figure 2:**
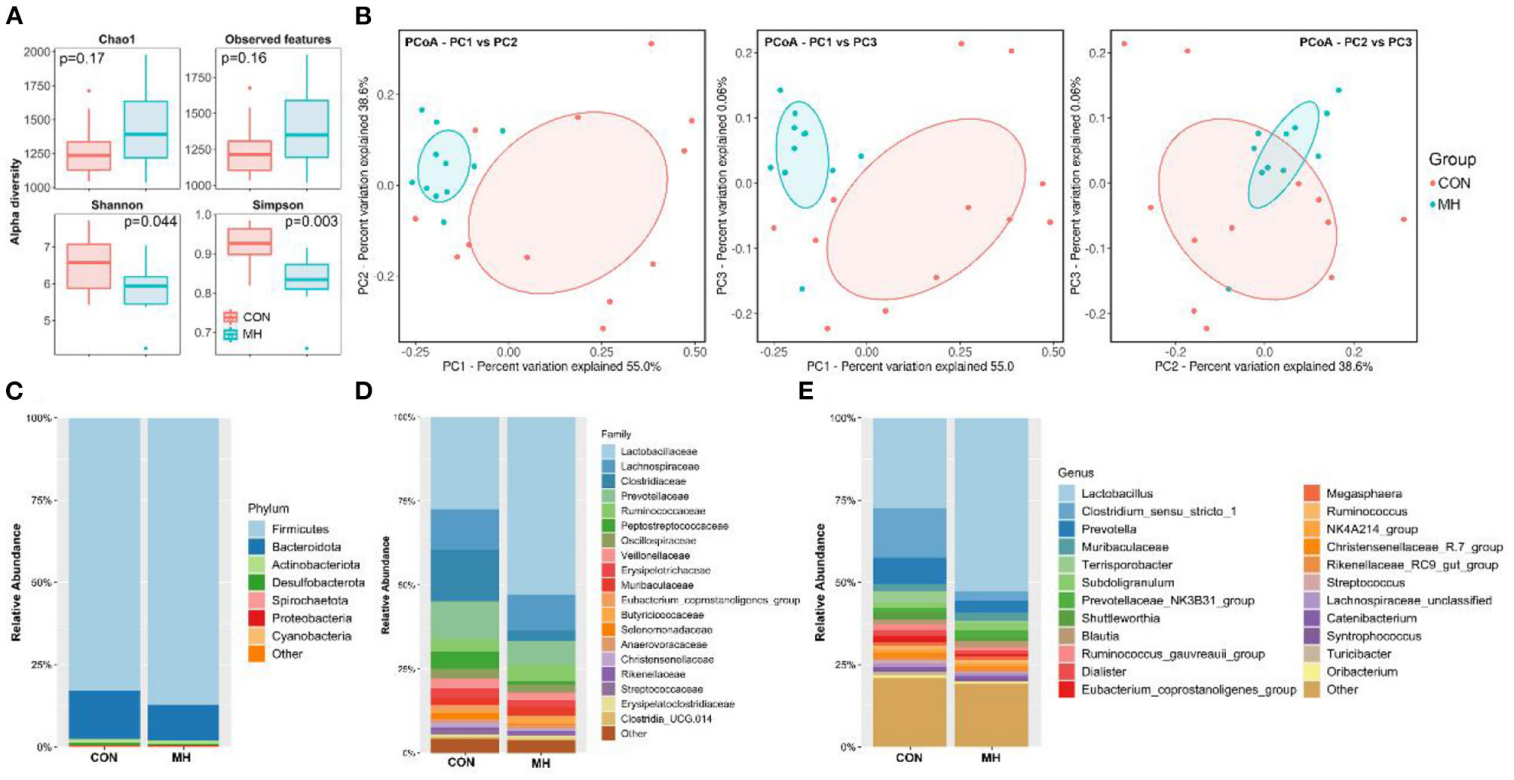
Fecal microbiota richness and evenness, difference of microbiome structure on β-diversity, and relative abundance of microbiota at phylum, family, and genus level. **(A)** Comparison of α-diversity indices, **(B)** comparison of β-diversity based on Principal coordinate analysis (PCoA), relative abundances at **(C)** phylum **(D)** family, and **(E)** genus levels. CON = basal diet without any additives, MH = basal diet + 8.3 × 10^9^ CFU/kg LM1, *n* =12 in the CON group, *n* =11 in the MH group.

The sequencing analysis of the 23 fecal samples yielded 5,775,046 total reads after filtering. According to the 97% similarity level, the CON group had 1037.92 ± 129 OTUs, whereas the MH treatment group had 984.91 ± 154 OTUs. The species richness (Chao1, observed - features) and diversity (Shannon and Simpson) indices were measured to verify the effects of probiotic supplementation on alpha diversity. The rarefaction curves for Chao1 and observed features (Supplementary Figure S1) tended to reach a plateau, thereby suggesting that a 40,000-sequencing depth was enough to capture the majority of the OTUs in the samples. Although the Chao1 index and the number of observed features did not exhibit any significant difference between the two groups. The MH treatment group had decreased population diversity and evenness than the CON group, as revealed by Shannon (*P* = 0.044) and Simpson (*P* = 0.003) indices, respectively (Figure 2A). Additionally, a PCoA plot was constructed, based on Bray-Curtis dissimilarity matrix, to investigate the changes in the gut microbiome structure between the CON and MH treatment groups (Figure 2B). The PCoA plot revealed that the gut microbiota of the MH-treated pigs had a significantly distinct cluster from that of the control pigs (*P* < 0.05), thereby suggesting that supplementation of diet with moderate-high dose of LM1 altered the gut microbiome of growing pigs.

### Effect of Dietary LM1 on the Gut Microbiota Composition of Growing Pigs

The LM1-mediated changes in the microbiota composition were investigated in this study (Figures 2C–E). With respect to the microbes at the phylum level, we examined the top 7 phyla with the higher relative abundance (Figure 2C, Supplementary Table S1). Bacteria belonging to the phyla *Firmicutes* and *Bacteroidetes* accounted for approximately 97% of the observed specimens in both CON and MH-treated groups, followed by bacteria belonging to *Actinobacteria* (1.21–1.31%), *Desulfobacterota* (0.60–0.63%), *Spirochaetota* (0.26–0.27%), *Proteobacteria* (0.14%), and *Cyanobacteria* (0.06%). Additionally, the proportion of *Firmicutes* was significantly elevated (*P* < 0.001), while that of *Bacteroidota* was significantly reduced (*P* < 0.001) in the MH treatment group, as compared to that in the control group.

At the family level, among the top 19 bacterial families (relative abundance > 0.1% in all samples), *Lactobacillaceae* (52.80%; *P* < 0.001), *Ruminococcaceae* (5.04%; *P* = 0.026), and *Butyricoccaceae* (1.76%; *P* = 0.028) were drastically enriched by MH supplementation. Conversely, the abundance of the bacterial families *Clostridiaceae* (2.97%; *P* = 0.009), *Peptostreptococcaceae* (0.83%; *P* = 0.007), *Eubacterium_ coprostanoligenes_*group (0.64%, *P* = 0.050), and *Selenomonadaceae* (0.51%; *P* = 0.026) were significantly decreased in the MH treatment group, as compared to that in the CON group (Figure 2D, Supplementary Table S2).

At the genus level (relative abundance >0.1% in all samples), *Lactobacillus, Clostridium_sensu_stricto_*1, and *Prevotella* were the major genera in the two groups. *Lactobacillus* (52.80%; *P* < 0.001) and *Subdoligranulum* (2.29%; *P* = 0.028) were significantly enriched, while *Clostridium_sensu_stricto_*1 (2.72%; *P* = 0.008), *Prevotella* (3.67%; *P* = 0.040)*, Eubacterium_ coprostanoligenes_*group (0.63%; *P* = 0.05), *Streptococcus* (0.49%; *P* = 0.050)*, Terrisporobacter* (0.05%; *P* = 0.004), and *Turicibacter* (0.16%; *P* = 0.017) were significantly reduced in the MH treatment group, as compared to that in the CON group (Figure 2E).

**Figure 3:**
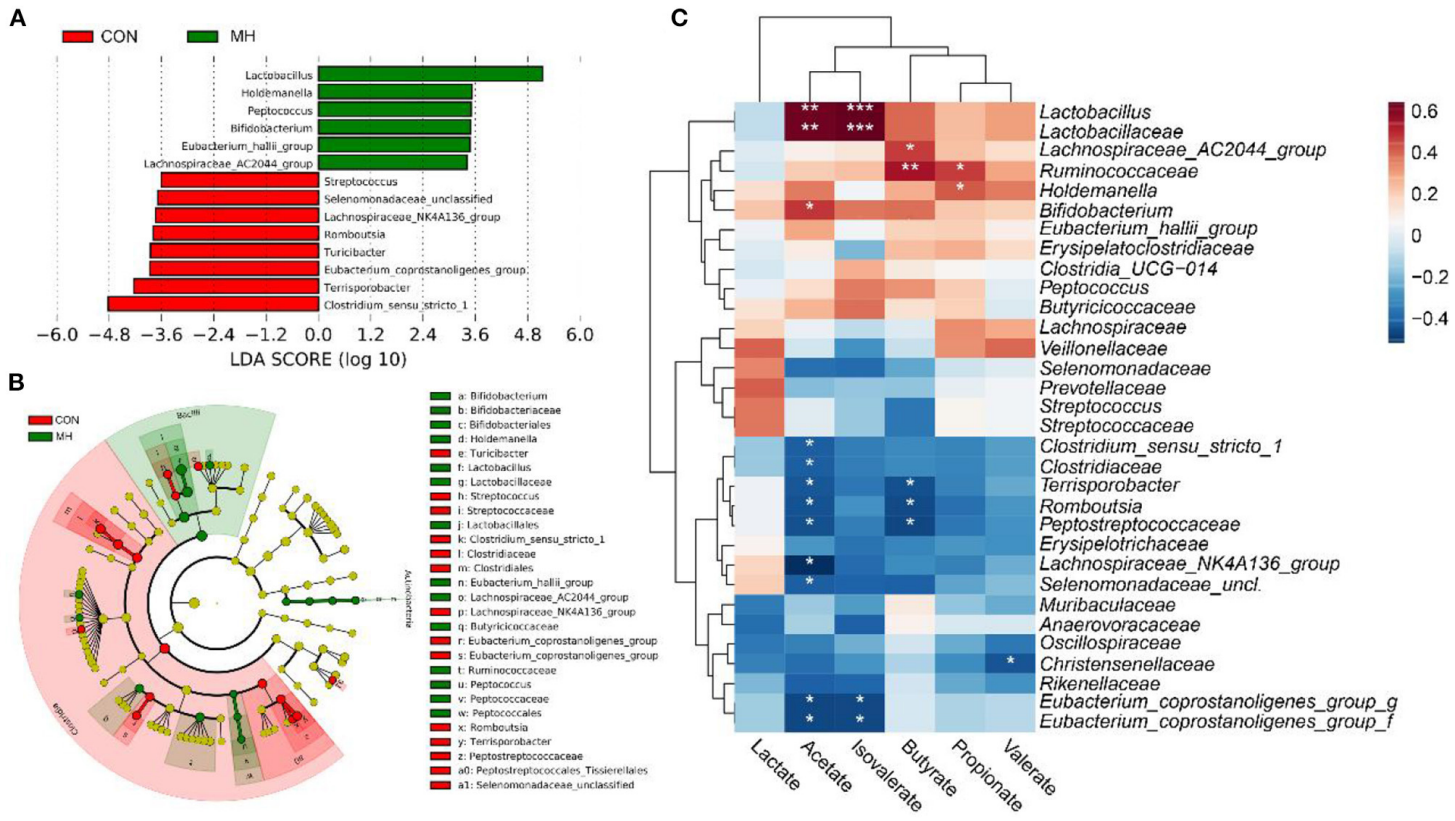
LefSE analysis of gut microbiota and correlation between characteristic bacteria and SCFA of growing pigs between 2 groups. **(A)** Linear discriminant analysis (LDA) score of the gut microbiota. **(B)** Cladogram of LEfSe shows taxonomic profiling at the family and genus levels, **(C)** Correlation between the gut microbiota and SCFA levels. CON = basal diet without any additives, MH = basal diet + 8.3 × 10^9^ CFU/kg LM1, *n* = 12 in the CON group, *n* = 11 in the MH group. *, **, and *** represented *P* < 0.05, 0.01, and 0.001, respectively.

To investigate the differential taxonomic markers in the pig gut, LEfSe analysis was performed (Figures 3A,B). The results revealed that bacterial genera, such as *Lactobacillus, Holdemanella, Peptococcus, Bifidobacterium, Eubacterium_hallii*_ group, and *Lachnospiraceae_AC2044_* group, were significantly enriched due to the probiotic supplementation (*P* < 0.05). Moreover, *Clostridium_sensu_stricto_1, Terrisporobacter, Eubacterium_ coprostanoligenes_* group*, Turicibacter, Romboutsia, Lachnospiraceae_NK4A136_*group, *Selenomonadaceae_unclassified*, and *Streptococcus* were significantly decreased (*P* < 0.05) in the MH treatment group, as compared to that in the CON group. These results indicate that moderate-high dose LM1 supplementation modulates the gut microbiome of the pigs and leads to the enrichment of specific taxa.

### Effect of Dietary LM1 on the Fecal SCFA Concentrations of Growing Pigs

As shown in Table 7, MH supplementation in the diet tended to elevate butyrate concentrations (*P* = 0.053) and significantly elevated fecal isovalerate concentrations (*P* = 0.040); however, it had no impact on acetate, propionate, valerate, or lactate concentrations (*P* > 0.05).

**Table 7:** Effects of moderate-high dose LM1 on fecal SCFA levels of growing pigs^*a*^ (μ mol/g dry feces).

**Items**	**CON**	**MH**	***P*-value**
Acetate	23.53 ± 1.26	26.42 ± 1.36	0.151
Propionate	22.57 ± 2.25	22.22 ± 2.44	0.919
Butyrate	13.96 ± 1.93	20.2 ± 2.10	0.053
Isovalerate	27.36 ± 1.29	31.88 ± 1.41	0.040
Valerate	2.98 ± 0.50	2.25 ± 0.55	0.346
Lactate	4.28 ± 0.44	3.37 ± 0.44	0.182

^a^*Value means ± standard error*.

### Correlation Between Gut Microbiota and SCFA Concentrations

The Spearman correlation coefficient was employed to examine whether any associations existed between the LM1-modulated microbiota and the altered SCFA levels. The results demonstrated that *Lactobacillus* and *Lactobacillaceae* had positive correlations with both the acetate (*R* = 0.63, *P* = 0.01, both) and isovalerate concentrations (*R* = 0.64, *P* < 0.001, both), and *Bifidobacterium* (*R* = 0.48, *P* = 0.022) also contributed to acetate production (Figure 3C). Furthermore, *Holdemanella* (*R* = 0.42, *P* = 0.048) and *Ruminococcaceae* (*R* = 0.47, *P* = 0.023) demonstrated significantly positive associations with propionate concentration, while *Ruminococcaceae* (*R* = 0.54, *P* = 0.007) and *Lachnospiraceae_AC2044_* group (R = 0.54, *P* = 0.029) were also positively linked to butyrate production. However, the SCFA levels, especially acetate, were negatively correlated with *Clostridium_sensu_stricto_1* (*R* = −0.42, *P* = 0.049), *Clostridiaceae* (R = −0.42, *P* = 0.049), *Terrisporobacter* (*R* = −0.43, *P* = 0.040)*, Romboutsia* (R = −0.46, *P* = 0.029)*, Peptostreptococcaceae* (*R* = −0.45, *P* = 0.033), *Lachnospiraceae_NK4A136_*group (*R* = −0.52, *P* = 0.035), *Selenomonadaceae*_*unclassified* (*R* = −0.42, *P* = 0.048), *Eubacterium_coprostanoligenes_*group*-*g-f (*R* > −0.5, *P*<*0.05*). Additionally, butyrate level was negatively correlated with *Terrisporobacter* (*R* = −0.44, *P* = 0.033)*, Romboutsia* (*R* = −0.46, *P* = 0.029), *and Peptostreptococcaceae* (*R* = −0.47, *P* = 0.024). Isovalerate level was negatively associated with *Eubacterium_coprostanoligenes_* group*-*g-f (*R* = −0.46, *P* = 0.027) and valerate concentration was negatively linked to *Christensenellaceae* (*R* = −0.44, *P* = 0.04). These results suggest a potential mechanism by which dietary supplementation with moderate-high dosage LM1 altered the host's SCFA production by modulating the gut microbiota.

## Discussion

To the best of our knowledge, this is the first study to analyze the effects of *Lm. mucosae* LM1 supplementation in the diet of growing pigs. The observations reveal that the dietary inclusion of increasing concentrations of LM1 did not improve the growth performance of the growing pigs during the 42-d feeding trial. Since ADFI is positively associated with backfat thickness and ADG, the observation suggests that the LM1 supplementation failed to improve feed intake of growing pigs. However, LM1 supplementation improved the ATTD of N, which, in turn, may be associated with the differential intestinal characteristics and alteration of the gut microbial community.

The gastrointestinal tract is the main site for nutrient absorption, which is influenced by the intestinal morphology, specifically VH, CD, and V/C ratio (23, 24). In the present, the MH-treated pigs had a higher VH in the jejunum and ileum as well as a greater V/C ratio in the ileum, as compared to that in the CON group. Previously, *L. rhamnosus* GG supplementation has been found to improve the mucosal barrier in weaning piglets (25). Therefore, the high value of VH in the MH-treated pigs may be due to the antimicrobial action of LM1 that prevents the adhesion of pathogenic bacteria (26) Consequently, higher villi adhered by multiple epithelial cells and may accompany with the improvement of brush-border digestive enzymes (27) which help in absorbing nutrients (25), may contribute to the high ATTD of N in these pigs. Interestingly, the increased V/C ratio in response to MH supplementation is a biomarker of improved absorptive capacity, as reported by Li et al. (28). Hence, the two indices may partially explain the higher ATTD of N induced in the MH-treated pigs, as compared to that in the control pigs.

Oxidative stress has an adverse impact on the organs of the body because excessive free radicals could damage the proteins as well as DNA in cells (29). Notably, the intestine is a site for oxidative stress reactions (30). In the current study, the oxidant status in the jejunal and ileal mucosa was analyzed using typical biomarkers, such as T-AOC and TBARS. T-AOC is a non-enzymatic antioxidant defense system that removes excess free radicals and prevents lipid peroxidation (16), while TBARS, a product of lipid peroxidation, reflects the degree of fat oxidation in cells as well as the damage to the cells (10). In this study, the MH-treated pigs exhibited a tendency to express reduced TBARS concentration in the ileal mucosa. Similar reports have confirmed the ability of *Lm. mucosae* to upregulate the mRNAs of antioxidant enzymes, such as superoxide dismutase, catalase, and glutathione peroxidase, thereby decreasing the lipid peroxidation in inflamed colonic tissues of pigs and rats (9, 10, 12). As reported by Wang et al. (31), probiotics has unique antioxidant system and stimulates the antioxidant response of the host. Hence, the attenuated TBARS possibly reflects the protective effect of MH in the gut and is corrected with better intestinal morphology.

Inflammation is a common clinical-pathological process, reflecting the host's struggle against antigens, which mainly involves pro-inflammatory and anti-inflammatory cytokines. However, hypersecretion of these cytokines can induce multiple organ dysfunction syndromes (32). While TNF-α, IL-1β, and IL-6 are pivotal pro-inflammatory cytokines that trigger immune responses, IL-10 is an anti-inflammatory cytokine that inhibits immune responses (33). In this study, IL-1β and TNF-α concentrations were linearly diminished, whereas IL-10 concentration was linearly augmented with the increasing doses of LM1 supplementation in the diet. Analogous results of reduced serum concentrations of IL-1β, IL-6, and TNF-α have been reported in rats with mild inflammation who were fed on a mixed *Limosilactobacillus fermentum* diet and as well as in lipopolysaccharide-challenged weaning pigs whose diets were supplemented with *L. salivarius* and *L. mucosae* AN1 (9, 10, 34, 35). Low et al. (36) have reported that IL-1β responds to microbial invasion as well as tissue damages and rapidly stimulates immune responses by activating lymphocytes or inducing the release of other cytokines. Additionally, TNF-α stimulates systemic inflammation and early phase reactions (37). On the contrary, IL-10 inhibits immune responses to maintain host homeostasis (10). The observed LM1- induced changes in the serum inflammatory factors can improve the host's immune response.

Within the entire gastrointestinal tract of pigs, the colon is the primary location for fiber fermentation because it is the most enriched in microorganisms (38). According to previous research, dietary probiotics can improve intestinal health because of their ability to modulate the gut microbiota (39). In this study, the probiotic supplementation decreased the Shannon and Simpson indices and significantly modulated the gut microbial composition, as revealed by PCoA. This is consistent with a previous study that reported decrease in species diversity in the fecal samples of laying hens whose diet had been supplemented with *Lm. oris* BSLO 180 (40). Moreover, PCoA verified that LM1 supplementation modulated the microbial species composition in the gut; in fact, a similar report has been described in case of oral administration of *L. casei* in mice (41).

After 42 d of dietary supplementation with MH, pigs exhibited an increased abundance of phylum *Firmicutes*, but a decreased level of phylum *Bacteroidetes*. Even though both these phyla are predominant in mammals, the energy absorption by the host is positively interrelated with an increment in the *Firmicutes* population rather than the elevation of the *Bacteroidetes* population (42). Although abundant *Bacteroidetes* contributes to low backfat thickness and light bodyweight (43), these two indices were not affected by the altered abundance of *Bacteroidetes* in this study. Specifically, dietary supplementation with LM1 modulated the abundance of bacterial families. Among them, *Lactobacillaceae, Ruminococcaceae*, and *Butyricoccaceae*, all of which belong to phylum *Firmicutes*, which specializes in decomposing fibers and producing lactate and SCFA (44). The positive correlation between *Lactobacillaceae* and the ATTD of N and GE, as reported by Le Sciellour (45), may explain the elevated ATTD of N observed in the LM1 groups. However, the *Peptostreptococcaceae, Selenomonadaceae*, and *Erysipelotrichaceae* populations were sharply reduced after LM1 supplementation for 42 d. The decrease of pathogenic *Peptococcus*, which causes various purulent infections (46), may reflect the capacity of LM1 with respect to modulation of the microbial population.

At the genus level, there was a drastic enrichment of *Lactobacillus* in the gut of MH pigs. Additionally, *Terrisporobacter, Eubacterium_hallii _*group*, Holdemanella, Bifidobacterium, Peptococcus*, and *Lachnospiraceae_AC2044_*group were also abundant. *Lactobacillus* and *Bifidobacterium* can produce large amounts of acetate (47, 48), which can be converted to butyrate by butyrate-producing bacteria, such *Eubacterium_hallii_*group and *Lachnospiraceae*, via a metabolite cross-feeding mechanism (49, 50). However, the exact function of *Terrisporobacter* is not clear. It can be related to body weight and serum lipid indices in older Korean woman, and low birthweight of infants fed formula diet (14, 51). Reportedly, *Lactobacillus* also metabolizes amino acids into isovalerate (52), and this is consistent with the elevated isovalerate levels in the MH-treated pigs of our study. *Peptococcus* can contribute to butyrate production via metabolism of peptones and amino acids (53). Interestingly, MH treatment reduced the abundance of *Clostridium_sensu_stricto_*1, which causes epithelial inflammation (3), as well as that of *Prevotella, Eubacterium_ coprostanoligenes_*group, and *Romboutsia*. As reported, *Prevotella* can ferment plant non-starch polysaccharides to SCFA and produce enzymes that degrade polysaccharides (3). The abundance of *Eubacterium_coprostanoligenes_* group is associated with the lowering of plasma cholesterol (54). Furthermore, *Romboutsia* is enriched in the healthy gut (55). Therefore, the results revealed that the addition of probiotic LM1 shapes the gut microbial structure of the pigs, which, in turn, may affect their metabolite concentrations.

Gut microorganisms play a vital role in SCFA production because they possess genes encoding enzymes that can degrade plant polysaccharides (56). Therefore, alterations in the gut microbiome may influence SCFA concentration. In this study, LM1 supplementation led to an increase in the butyrate and isovalerate levels in the pigs. Butyrate is the main energy source for enterocytes, and it has a role in maintaining intestinal homeostasis by suppressing the proliferation of pro-inflammatory cytokines (57, 58). In a recent study, Zhong et al. (59) has demonstrated that gut microbial-produced butyrate is correlated with reduced intestinal inflammation and improved gut health in post-weaning pigs. Furthermore, increased isovalerate levels may indicate an increase in protein fermentation by the gut microbiota (60). According to the correlation analysis, *Ruminococcaceae* was positively associated with the increase in butyrate levels, while *Eubacterium_coprostanoligenes_*group was negatively correlated with butyrate levels. Additionally, isovalerate production was positively correlated with *Lactobacillaceae and Lactobacillus*, but it was negatively linked to *Eubacterium_coprostanoligenes_*group. Hence, it is speculated that the changes in the gut microbiota of LM1-treated pigs contributed to their fermentation ability more than that in the control pigs.

## Conclusion

In conclusion, dietary supplementation of LM1 in growing pig's diet at increasing doses did not affect the growth performance and backfat thickness. Nevertheless, the ATTD of N was improved, which may be linked to the increase in the jejunal villus height. Additionally, the proinflammatory factors, such as IL-1β and TNF-α, were suppressed and anti-inflammatory cytokine IL-10 was enhanced in the serum, thereby indicating a modulation of the immune response. Furthermore, the supplementation of LM1 enriched the SCFA-producing taxa, such as *Lactobacillus, Holdemanella, Peptococcus, Bifidobacterium, Eubacterium_hallii_*group, which ultimately affected the microbial metabolites, particularly butyrate and isovalerate. To our knowledge, this study is the first to evaluate the probiotic potential of novel probiotic *Lm. mucosae* LM1 *in vivo*. The results of the current study offer valuable insights on the application of LM1 on livestock animals, especially on pigs. Further investigation might be necessary to fully verify the beneficial effects of LM1 on pig's health.

## Data Availability Statement

All standard sequence format (.fastq) files generated by Illumina Miseq containing all raw sequence reads have been deposited at the National Center for Biotechnology Information (NCBI) Sequence Read Archive (SRA) (BioProject accession number: PRJNA826560).

## Ethics Statement

This work was supported by Korea Institute of Planning and Evaluation for Technology in Food, Agriculture, Forestry and Fisheries (IPET) through the Technology Commercialization Support Program, funded by the Ministry of Agriculture, Food and Rural Affairs (MAFRA). (Grant No: 122038-2).

## Author Contributions

QZ and RV analyzed the data and wrote the manuscript. JY and SK finished the animal experiment and helped to detect indices. DK designed the experiment and revised the manuscript. IK designed the experiment, provided the funds, and revised the manuscript. All authors contributed to the article and approved the submitted version.

## Funding

This work was supported by Korea Institute of Planning and Evaluation for Technology in Food, Agriculture, Forestry and Fisheries (IPET) through the Technology Commercialization Support Program, funded by the Ministry of Agriculture, Food and Rural Affairs (MAFRA). (Grant No: 122038-2).

## Conflict of Interest

The authors declare that the research was conducted in the absence of any commercial or financial relationships that could be construed as a potential conflict of interest.

## Publisher's Note

All claims expressed in this article are solely those of the authors and do not necessarily represent those of their affiliated organizations, or those of the publisher, the editors and the reviewers. Any product that may be evaluated in this article, or claim that may be made by its manufacturer, is not guaranteed or endorsed by the publisher.
