# Supplementary material for: Dietary Supplementation of Limosilactobacillus mucosae LM1 Enhances Immune Functions and Modulates Gut Microbiota Without Affecting the Growth Performance of Growing Pigs
Source: Front Vet Sci. 2022 Jun 30;9:918114. doi: 10.3389/fvets.2022.918114 (PMC9280434; doi:10.3389/fvets.2022.918114)
Supplement: Supplementary file 1 [file Data_Sheet_1.PDF]

Dietary Supplementation of *Limosilactobacillus mucosae* LM1 Enhances Immune Functions and Modulates Gut Microbiota without Affecting the Growth Performance of Growing Pigs

SUPPLEMENTARY DATA

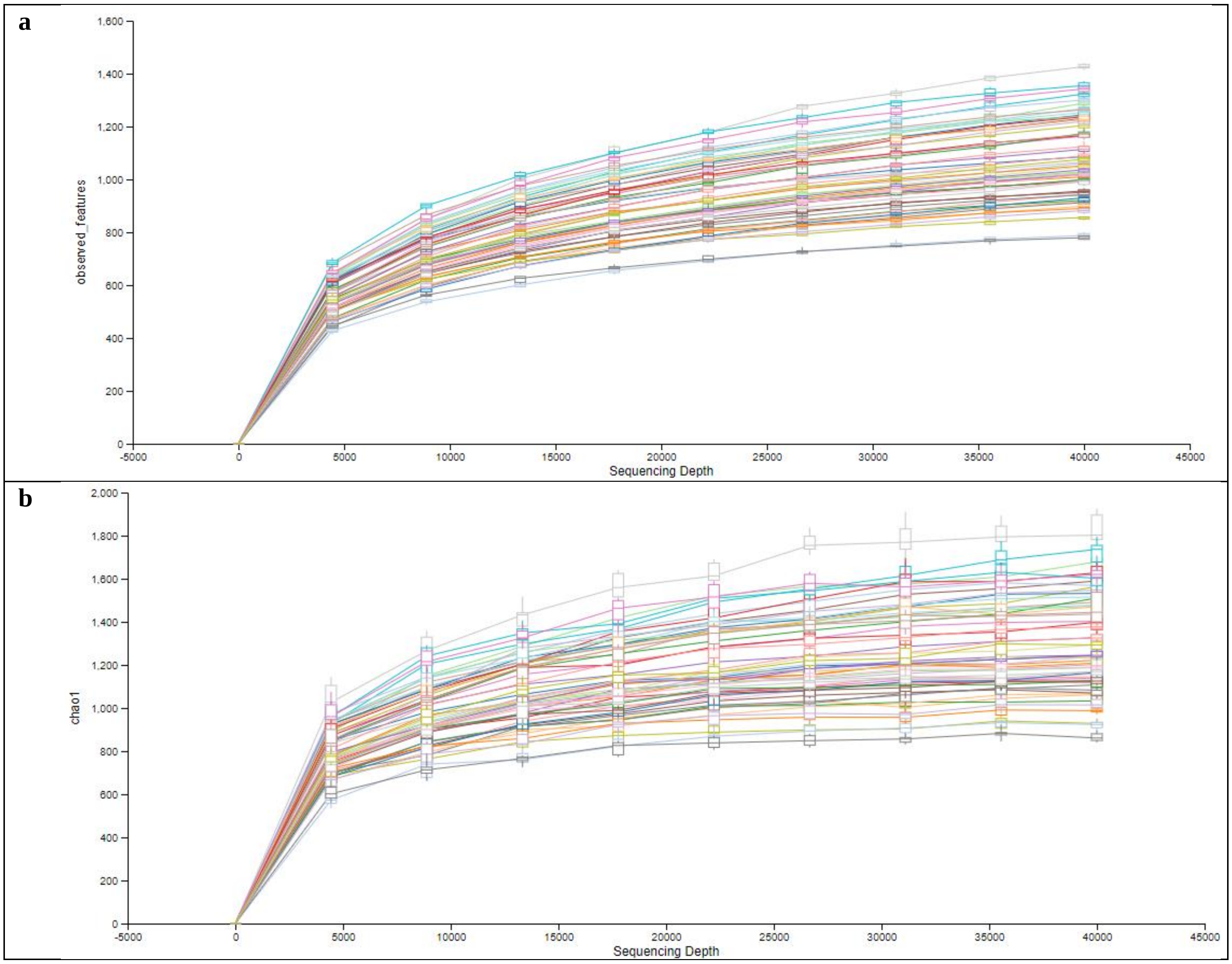

Figure S1. Rarefaction curves (a) Observed features and (b) Chao1 at 40000 sequencing depth.

Table S1. Relative abundances of taxonomic groups at phylum level. Cut-off set at 0.1%.

Phyla	Relative abundance (%)		*P*-value
	CON (n=12)	MH (n=11)	
Firmicutes (Bacillota)	82.95 ± 6.25	84.13 ± 5.87	< 0.001
Bacteroidetes (Bacteroidota)	14.57 ± 6.02	13.32 ± 5.64	< 0.001
Actinobacteria (Actinomycetota)	1.21 ± 1.13	1.31 ± 0.46	0.83
Desulfobacterota	0.63 ± 0.80	0.60 ± 0.26	0.71
Spirochaetota	0.27 ± 0.29	0.26 ± 0.34	0.92
Proteobacteria (Pseudomonadota)	0.14 ± 0.19	0.14 ± 0.20	0.96
Cyanobacteria	0.06 ± 0.05	0.06 ± 0.12	0.97
Verrucomicrobiota	0.07 ± 0.11	0.07 ± 0.04	0.95
Planctomycetota	0.03 ± 0.04	0.03 ± 0.04	0.99
Fibrobacterota	0.04 ± 0.04	0.03 ± 0.01	0.98
Campilobacterota	0.04 ± 0.07	0.04 ± 0.01	0.96
Other phyla	0.01 ± 0.01	0.01 ± 0.04	

Values were reported as mean ± standard deviation (SD).
P-values were calculated using Welch's t-test with false discovery rate (FDR) correction.
CON, basal diet without any additives; MH, basal diet + 8.3×10^9 CFU/kg LM1
Some of the new validly published phylum names are noted in parentheses.

Table S2. Relative abundances of taxonomic groups at family level. Cut-off set at 0.1%.

**Families**	**Relative abundance (%)**		***P*-value**
	CON (n=12)	MH (n=11)	
Lactobacillaceae	27.40 ± 17.58	52.80 ± 11.93	< 0.001
Lachnospiraceae	12.21 ± 4.77	10.82 ± 3.01	0.42
Clostridiaceae	15.23 ± 13.87	2.97 ± 2.66	0.009
Prevotellaceae	11.27 ± 6.83	7.11 ± 4.52	0.10
Ruminococcaceae	3.68 ± 1.43	5.04 ± 1.29	0.03
Peptostreptococcaceae	5.13 ± 4.70	0.83 ± 0.72	0.007
Oscillospiraceae	2.85 ± 2.33	2.42 ± 1.46	0.61
Veillonellaceae	2.93 ± 2.12	2.23 ± 1.99	0.42
Erysipelotrichaceae	2.83 ± 1.22	2.05 ± 0.74	0.08
Muribaculaceae	2.15 ± 1.61	2.54 ± 1.86	0.60
Eubacterium coprostanoligenes group	1.87 ± 1.97	0.64 ± 0.21	0.05
Butyricicoccaceae	0.69 ± 0.73	1.76 ± 1.38	0.03
Selenomonadaceae	1.79 ± 1.67	0.51 ± 0.59	0.03
Anaerovoracaceae	1.07 ± 0.59	0.97 ± 0.32	0.63
Christensenellaceae	1.20 ± 1.67	0.67 ± 0.53	0.32
Rikenellaceae	0.94 ± 0.87	0.90 ± 0.98	0.92
Streptococcaceae	1.23 ± 1.21	0.50 ± 0.95	0.12
Erysipelatoclostridiaceae	0.74 ± 0.84	0.95 ± 0.62	0.51
Clostridia UCG-014	0.54 ± 0.34	0.60 ± 0.15	0.60
Other families	4.24 ± 2.95	3.68 ± 1.04	

Values were reported as mean ± standard deviation (SD).
P-values were calculated using Welch's t-test with false discovery rate (FDR) correction.
CON, basal diet without any additives; MH, basal diet + 8.3 × 10^9 CFU/kg LM1

Table S3. Relative abundances of taxonomic groups at genus level. Cut-off set at 0.1%.

**Genera**	**Relative abundance (%)**		***P*-value**
	CON (n=12)	MH (n=11)	
Lactobacillus	27.4 ± 17.57	52.8 ± 11.29	0.0005
Clostridium sensu stricto 1	15.0 ± 13.74	2.72 ± 2.505	0.008
Prevotella	8.12 ± 6.37	3.67 ± 2.30	0.040
Muribaculaceae	2.14 ± 1.61	2.53 ± 1.86	0.597
Terrisporobacter	3.67 ± 3.17	0.50 ± 0.51	0.003
Subdoligranulum	1.29 ± 0.82	2.29 ± 1.18	0.028
Prevotellaceae NK3B31 group	1.34 ± 1.30	2.11 ± 2.93	0.422
Shuttleworthia	2.19 ± 2.45	1.09 ± 0.84	0.172
Blautia	1.44 ± 1.41	1.80 ± 1.06	0.498
Ruminococcus gauvreauii group	1.91 ± 2.42	1.08 ± 0.62	0.285
Dialister	1.65 ± 1.20	1.09 ± 0.74	0.196
Eubacterium coprostanoligenes group	1.87 ± 1.95	0.63 ± 0.21	0.050
Megasphaera	1.24 ± 1.30	1.13 ± 1.44	0.841
Ruminococcus	0.95 ± 0.52	1.19 ± 0.77	0.386
NK4A214 group	1.10 ± 1.03	0.86 ± 0.57	0.500
Christensenellaceae R-7 group	1.19 ± 1.66	0.66 ± 0.52	0.318
Rikenellaceae RC9 gut group	0.92 ± 0.83	0.89 ± 0.96	0.944
Streptococcus	1.22 ± 1.20	0.49 ± 0.95	0.124
Lachnospiraceae unclassified	0.90 ± 0.40	0.82 ± 0.35	0.627
Catenibacterium	0.66 ± 0.83	0.88 ± 0.63	0.473
Syntrophococcus	0.77 ± 0.58	0.73 ± 0.48	0.866
Turicibacter	1.24 ± 1.36	0.15 ± 0.24	0.017
Oribacterium	0.74 ± 0.53	0.67 ± 0.32	0.691
Selenomonadaceae uncultured	1.10 ± 1.15	0.26 ± 0.46	0.034
Butyricicoccaceae unclassified	0.32 ± 0.44	1.07 ± 1.35	0.084
Oscillospiraceae UCG-002	0.70 ± 0.61	0.59 ± 0.43	0.630

Faecalibacterium	0.60 ± 0.72	0.67 ± 0.77	0.810
Coprococcus	0.54 ± 0.42	0.71 ± 0.21	0.253
Romboutsia	1.07 ± 1.43	0.09 ± 0.15	0.035
Clostridia UCG-014	0.54 ± 0.34	0.60 ± 0.15	0.601
Olsenella	0.75 ± 1.22	0.31 ± 0.26	0.248
Eubacterium hallii group	0.42 ± 0.31	0.59 ± 0.25	0.173
Solobacterium	0.44 ± 0.21	0.54 ± 0.30	0.356
Agathobacter	0.50 ± 0.56	0.46 ± 0.54	0.852
Oscillospiraceae UCG-005	0.46 ± 0.42	0.48 ± 0.42	0.924
Desulfovibrio	0.58 ± 0.79	0.30 ± 0.25	0.275
Prevotellaceae uncultured	0.51 ± 0.49	0.38 ± 0.22	0.412
Roseburia	0.51 ± 0.26	0.37 ± 0.48	0.371
Catenisphaera	0.40 ± 0.24	0.43 ± 0.26	0.764
Holdemanella	0.26 ± 0.18	0.57 ± 0.37	0.019
Prevotellaceae unclassified	0.55 ± 0.47	0.25 ± 0.29	0.081
Alloprevotella	0.36 ± 0.17	0.42 ± 0.49	0.710
Bifidobacterium	0.25 ± 0.16	0.45 ± 0.26	0.037
Lachnospiraceae NK3A20 group	0.41 ± 0.58	0.27 ± 0.08	0.420
Butyricicoccaceae UCG-008	0.21 ± 0.36	0.48 ± 0.41	0.107
Family XIII AD3011 group	0.33 ± 0.23	0.33 ± 0.20	0.977
Dorea	0.23 ± 0.17	0.40 ± 0.27	0.075
Oscillospirales UCG-010	0.33 ± 0.26	0.27 ± 0.14	0.501
Eubacterium nodatum group	0.38 ± 0.44	0.20 ± 0.15	0.226
Marvinbryantia	0.22 ± 0.16	0.30 ± 0.16	0.313
Ruminococcaceae uncultured	0.23 ± 0.13	0.28 ± 0.22	0.544
Family XIII UCG-001	0.22 ± 0.05	0.28 ± 0.11	0.087
Intestinibacter	0.27 ± 0.14	0.21 ± 0.11	0.288
Oscillospiraceae unclassified	0.25 ± 0.31	0.22 ± 0.07	0.711

Mitsuokella	0.30 ± 0.33	0.15 ± 0.21	0.248
Lachnospiraceae NK4A136 group	0.27 ± 0.14	0.15 ± 0.10	0.032
Phascolarctobacterium	0.16 ± 0.19	0.27 ± 0.24	0.241
Clostridium sensu stricto 6	0.18 ± 0.17	0.23 ± 0.23	0.537
Treponema	0.25 ± 0.29	0.16 ± 0.31	0.452
Incertae Sedis	0.17 ± 0.09	0.22 ± 0.13	0.337
Pseudoramibacter	0.31 ± 0.53	0.08 ± 0.13	0.189
RF39	0.16 ± 0.10	0.22 ± 0.11	0.178
Erysipelotrichaceae UCG-006	0.17 ± 0.15	0.18 ± 0.10	0.878
Peptococcus	0.11 ± 0.07	0.22 ± 0.16	0.047
Ruminococcaceae unclassified	0.19 ± 0.27	0.13 ± 0.14	0.477
Eubacterium ruminantium group	0.15 ± 0.17	0.16 ± 0.15	0.818
Selenomonadaceae unclassified	0.25 ± 0.24	0.05 ± 0.04	0.013
Prevotellaceae UCG-004	0.18 ± 0.25	0.09 ± 0.11	0.274
Bacteroidales uncultured	0.12 ± 0.06	0.14 ± 0.09	0.406
Monoglobus	0.13 ± 0.09	0.13 ± 0.06	0.989
Butyricicoccus	0.11 ± 0.14	0.14 ± 0.16	0.653
Erysipelotrichaceae UCG-009	0.21 ± 0.45	0.04 ± 0.04	0.250
Lachnospiraceae AC2044 group	0.03 ± 0.02	0.21 ± 0.29	0.039
Fusicatenibacter	0.09 ± 0.11	0.13 ± 0.18	0.461
Prevotellaceae UCG-003	0.09 ± 0.07	0.10 ± 0.15	0.752
Lachnospira	0.09 ± 0.06	0.09 ± 0.06	0.830
Acidaminococcus	0.14 ± 0.19	0.03 ± 0.04	0.068
Mogibacterium	0.08 ± 0.06	0.08 ± 0.04	0.944
Lachnospiraceae FCS020 group	0.07 ± 0.04	0.08 ± 0.02	0.728
Oscillibacter	0.08 ± 0.05	0.07 ± 0.08	0.611
Escherichia-Shigella	0.02 ± 0.03	0.13 ± 0.20	0.071
Ruminococcus torques group	0.04 ± 0.04	0.10 ± 0.08	0.032

Erysipelotrichaceae uncultured	0.06 ± 0.05	0.08 ± 0.06	0.301
Gastranaerophilales	0.05 ± 0.04	0.08 ± 0.11	0.478
Butyrivibrio	0.08 ± 0.04	0.05 ± 0.02	0.014
Collinsella	0.05 ± 0.04	0.08 ± 0.07	0.293
Prevotellaceae UCG-001	0.07 ± 0.10	0.06 ± 0.13	0.890
Candidatus Soleaferrea	0.06 ± 0.06	0.06 ± 0.05	0.883
Oscillospira	0.05 ± 0.03	0.07 ± 0.06	0.555
Lachnospiraceae ND3007 group	0.04 ± 0.05	0.07 ± 0.08	0.309
Peptostreptococcaceae uncl.	0.09 ± 0.06	0.01 ± 0.02	0.000
Lachnospiraceae NK4B4 group	0.07 ± 0.08	0.03 ± 0.03	0.108
Pseudoscardovia	0.05 ± 0.09	0.05 ± 0.06	0.887
Anaerovoracaceae unclassified	0.04 ± 0.04	0.06 ± 0.08	0.553
Colidextribacter	0.07 ± 0.09	0.03 ± 0.04	0.160
Lachnospiraceae FE2018 group	0.08 ± 0.20	0.01 ± 0.02	0.232
Eubacterium siraeum group	0.03 ± 0.02	0.06 ± 0.03	0.034
Selenomonas	0.08 ± 0.11	0.00 ± 0.00	0.056
Negativibacillus	0.04 ± 0.03	0.04 ± 0.02	0.970
Clostridium methylpentosum group	0.02 ± 0.04	0.06 ± 0.06	0.092
Eubacterium eligens group	0.03 ± 0.03	0.03 ± 0.02	0.918
Clostridia unclassified	0.04 ± 0.02	0.02 ± 0.00	0.120
Fournierella	0.03 ± 0.02	0.04 ± 0.02	0.322
Butyricicoccaceae UCG-009	0.02 ± 0.03	0.04 ± 0.02	0.290
Parabacteroides	0.01 ± 0.03	0.05 ± 0.06	0.105
Chlamydia	0.04 ± 0.09	0.01 ± 0.02	0.396
Succinivibrio	0.04 ± 0.08	0.01 ± 0.01	0.203
Erysipelatoclostridiaceae UCG-004	0.02 ± 0.02	0.03 ± 0.03	0.465
WPS-2	0 ± 0	0.06 ± 0.19	0.253
Eggerthellaceae uncultured	0.02 ± 0.02	0.04 ± 0.02	0.053

Enterorhabdus	0.02 ± 0.01	0.03 ± 0.02	0.068
Lachnospiraceae NC2004 group	0.03 ± 0.01	0.02 ± 0.01	0.503
Sharpea	0.04 ± 0.05	0.01 ± 0.02	0.116
Clostridia vadin BB60 group	0.02 ± 0.02	0.02 ± 0.02	0.963
Eubacterium xylanophilum group	0.02 ± 0.04	0.03 ± 0.04	0.777
p-2534-18B5 gut group	0.03 ± 0.11	0.02 ± 0.05	0.749
Peptococcaceae uncultured	0.02 ± 0.01	0.03 ± 0.02	0.210
p-1088-a5 gut group	0.02 ± 0.04	0.02 ± 0.03	0.966
Anaerovibrio	0.02 ± 0.03	0.02 ± 0.02	0.665
Bradymonadales	0.03 ± 0.03	0.01 ± 0.03	0.155
Eubacterium ventriosum group	0.01 ± 0.01	0.03 ± 0.04	0.248
Moryella	0.02 ± 0.01	0.02 ± 0.01	0.556
Fibrobacter	0.03 ± 0.04	0.01 ± 0.01	0.067
Lachnospiraceae XPB1014 group	0.00 ± 0.01	0.03 ± 0.07	0.223
Campylobacter	0.03 ± 0.06	0.00 ± 0.00	0.128
Eubacterium oxidoreducens group	0.01 ± 0.01	0.01 ± 0.00	0.767
Coriobacteriales uncultured	0.01 ± 0.01	0.01 ± 0.01	0.612
CAG-352	0.02 ± 0.06	0.00 ± 0.00	0.209
Lachnospiraceae UCG-004	0.01 ± 0.01	0.01 ± 0.01	0.620
dgA-11 gut group	0.02 ± 0.02	0.00 ± 0.01	0.134
Acetitomaculum	0.01 ± 0.02	0.01 ± 0.01	0.852
Lachnoclostridium	0.01 ± 0.02	0.01 ± 0.01	0.628
Sphaerochaeta	0.00 ± 0.00	0.02 ± 0.02	0.077
WCHB1-41	0.02 ± 0.03	0.00 ± 0.01	0.173
Lachnospiraceae uncultured	0.01 ± 0.01	0.01 ± 0.01	0.474
Succinivibrionaceae UCG-001	0.02 ± 0.05	0.00 ± 0.00	0.143
Allisonella	0.02 ± 0.02	0.00 ± 0.00	0.020
Bacteroides	0.01 ± 0.05	0.00 ± 0.00	0.357

Schwartzia	0.01 ± 0.02	0.00 ± 0.01	0.695
Senegalimassilia	0.01 ± 0.01	0.01 ± 0.01	0.999
Anaerorhabdus furcosa group	0.01 ± 0.01	0.01 ± 0.01	0.958
Lachnospiraceae UCG-008	0.00 ± 0.00	0.01 ± 0.01	0.040
Other genera	0.30 ± 0.15	0.25 ± 0.10	

Values were reported as mean ± standard deviation (SD).
P-values were calculated using Welch's t-test with false discovery rate (FDR) correction.
CON, basal diet without any additives; MH, basal diet + 8.3 × 10^9 CFU/kg LM1
